# Supplementary material for: CryoEM structure of the human SLC4A4 sodium-coupled acid-base transporter NBCe1
Source: Nat Commun. 2018 Mar 2;9:900. doi: 10.1038/s41467-018-03271-3 (PMC5834491; doi:10.1038/s41467-018-03271-3)
Supplement: Supplementary file 1 — Supplementary Information [file 41467_2018_3271_MOESM1_ESM.pdf]

Supplementary Information

CryoEM Structure of the Human SLC4A4 Sodium-Coupled Acid-Base Transporter NBCe1

Huynh et al.

Supplementary Figure 1

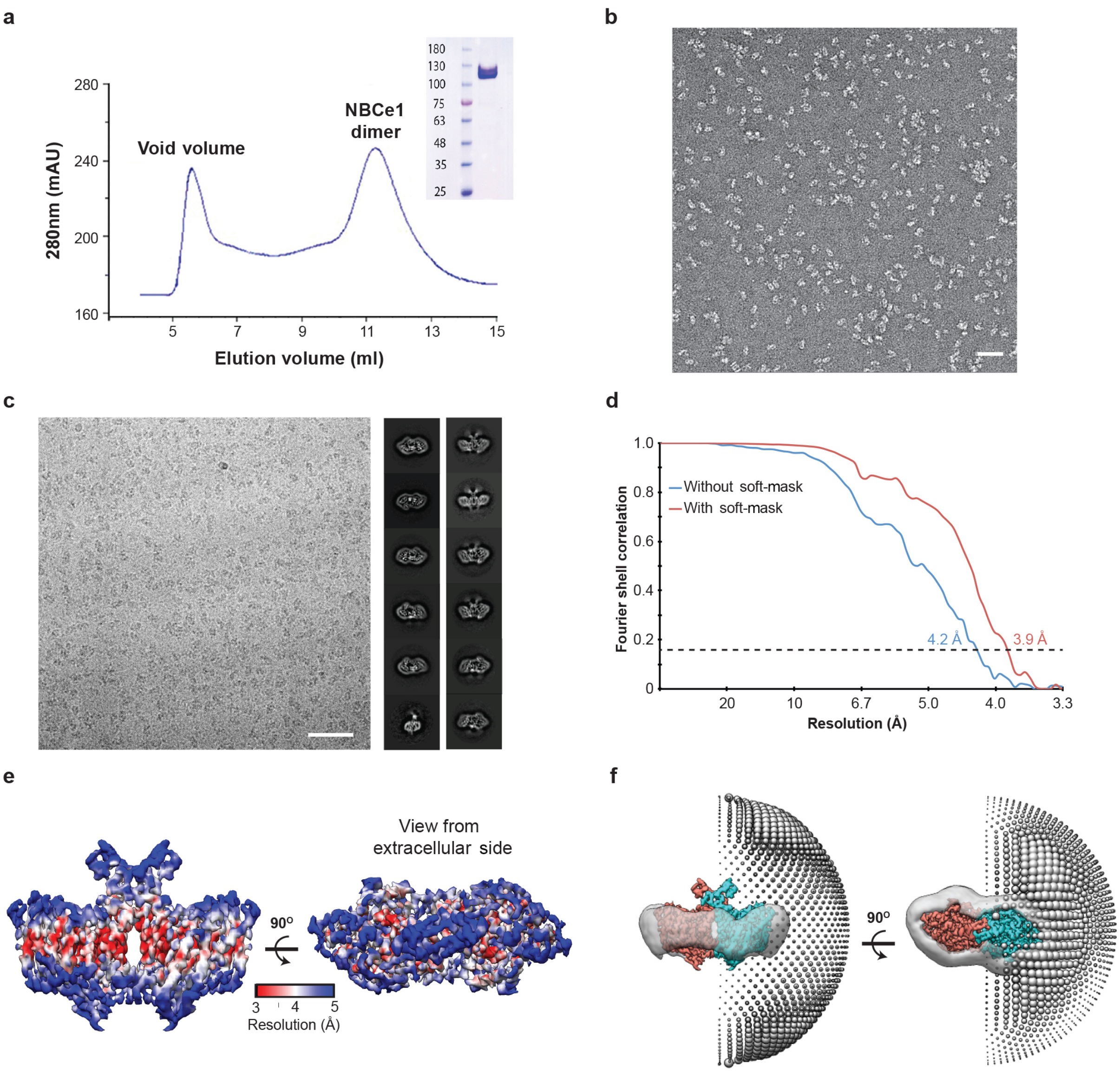

Supplementary Figure 1 Sample characterization and cryoEM image processing. a Size-exclusion chromatography on a Superose 6 column of the NBCe1 sample after affinity chromatography. A single symmetrical peak corresponds to the NBCe1 dimer. A small shoulder before the peak represents NBCe1 tetramers and higher oligomers (<3% of total NBCe1). SDS-PAGE of purified NBCe1 (top right). Two bands represent glycosylated (upper band) and non-glycosylated (lower band) forms. Size markers in kDa are shown on the left. **b** A representative negatively stained micrograph of NBCe1 (bar, 40 nm). **c** A representative cryoEM micrograph of NBCe1 (bar, 50 nm). Different views of representative 2D class averages are shown to the right. **d** Fourier shell correlation (FSC) curves for the final reconstruction of NBCe1 prior to and following post-processing in RELION-2. The final resolution was estimated using the FSC=0.143 criterion. **e** Local resolution analysis of the NBCe1 cryoEM map using ResMap. **f** Angular distribution map of NBCe1 post-processed structure.

Supplementary Figure 2

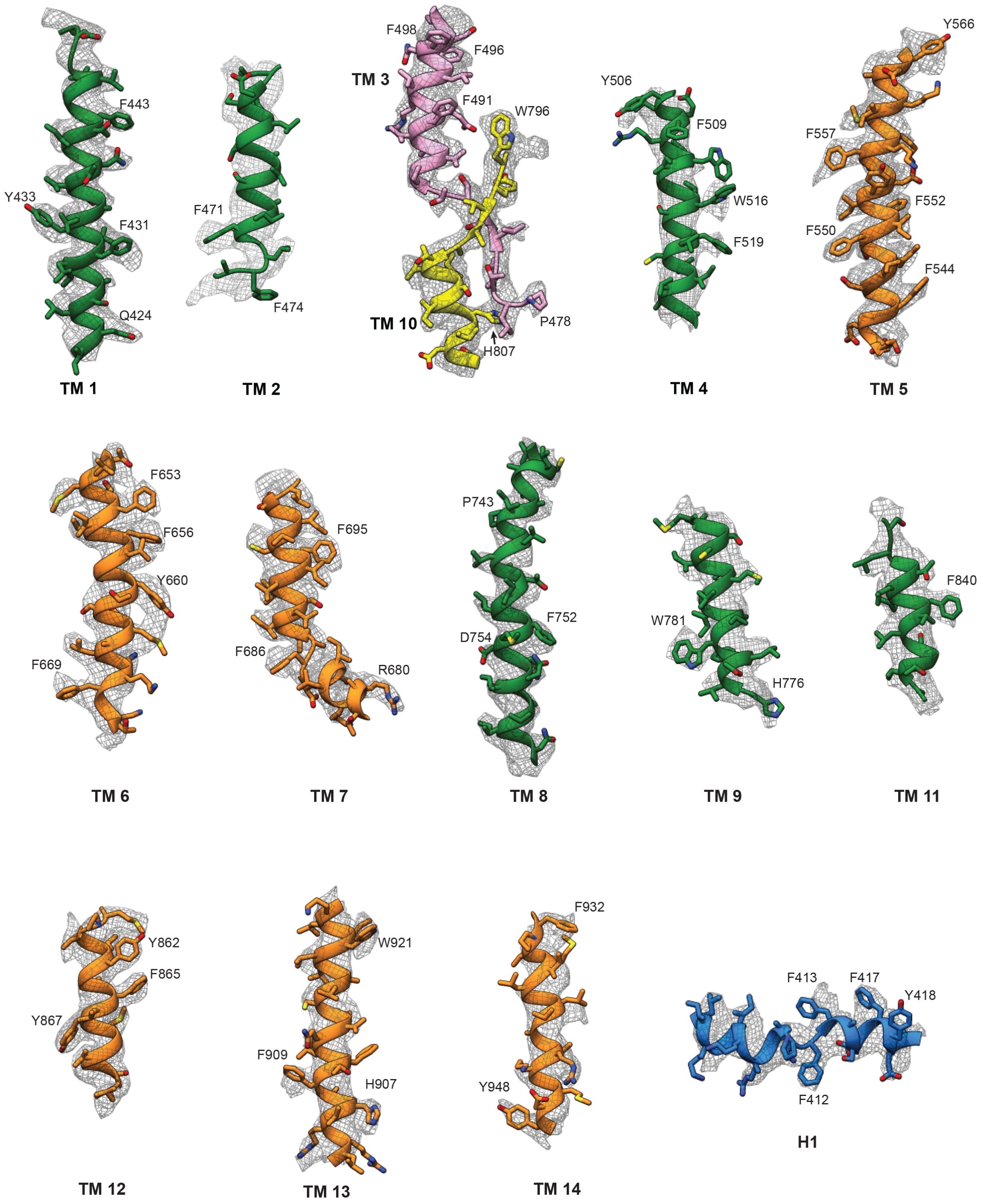

Supplementary Figure 2 Representative regions of the cryoEM density map (mesh) superposed with atomic models (ribbon). Bulky side chain residues are labeled.

Supplementary Figure 3

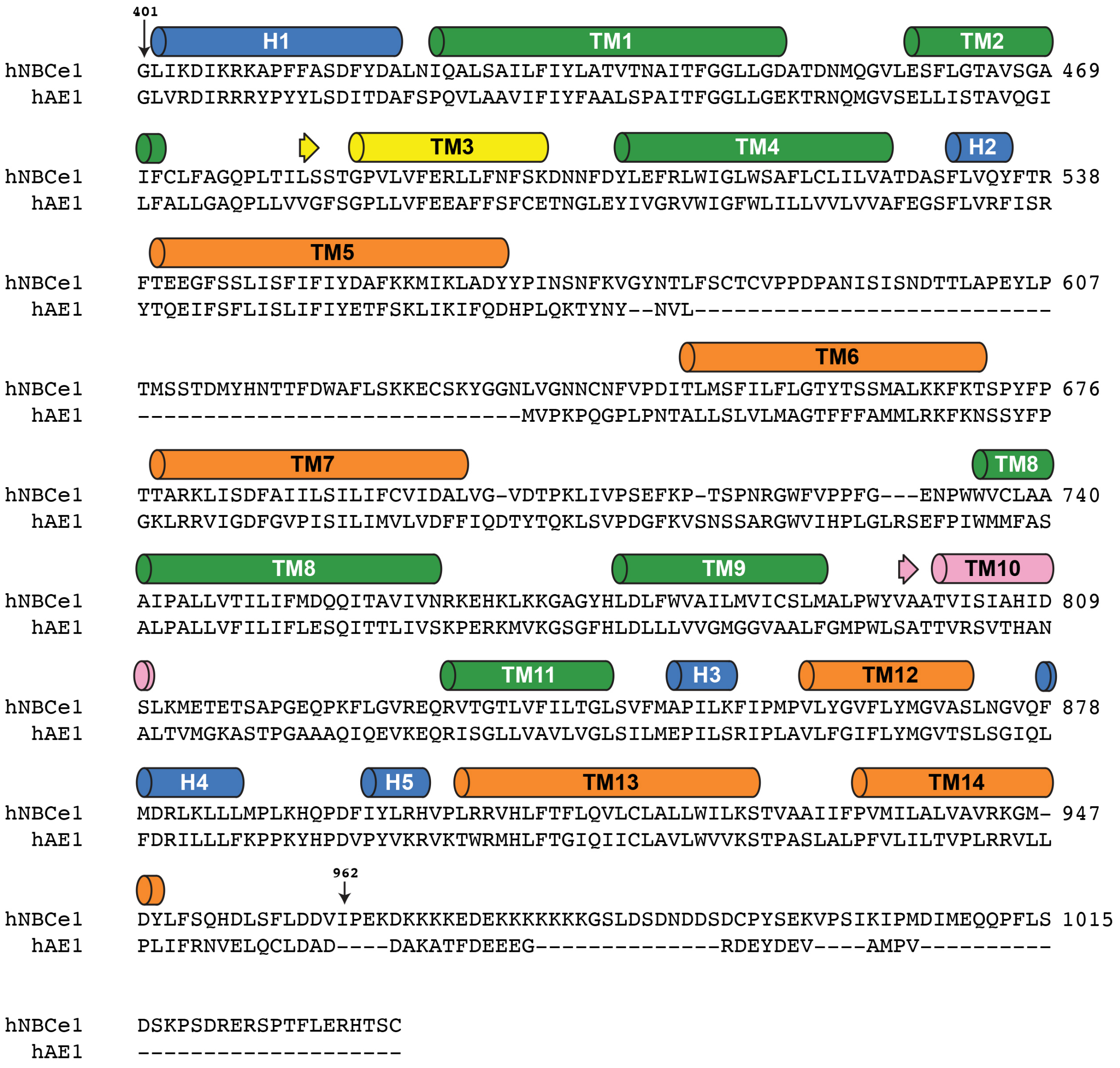

Supplementary Figure 3 Sequence alignment of human NBCe1 and AE1. The sequence alignment of the membrane domains of human NBCe1 and human AE1. Cylinders and arrows above the sequence represent α-helices and β-strands respectively. The coloring scheme is similar to Fig. 1c. The black arrows above the NBCe1 sequence indicate the first and last residues of our NBCe1 model.

Supplementary Figure 4

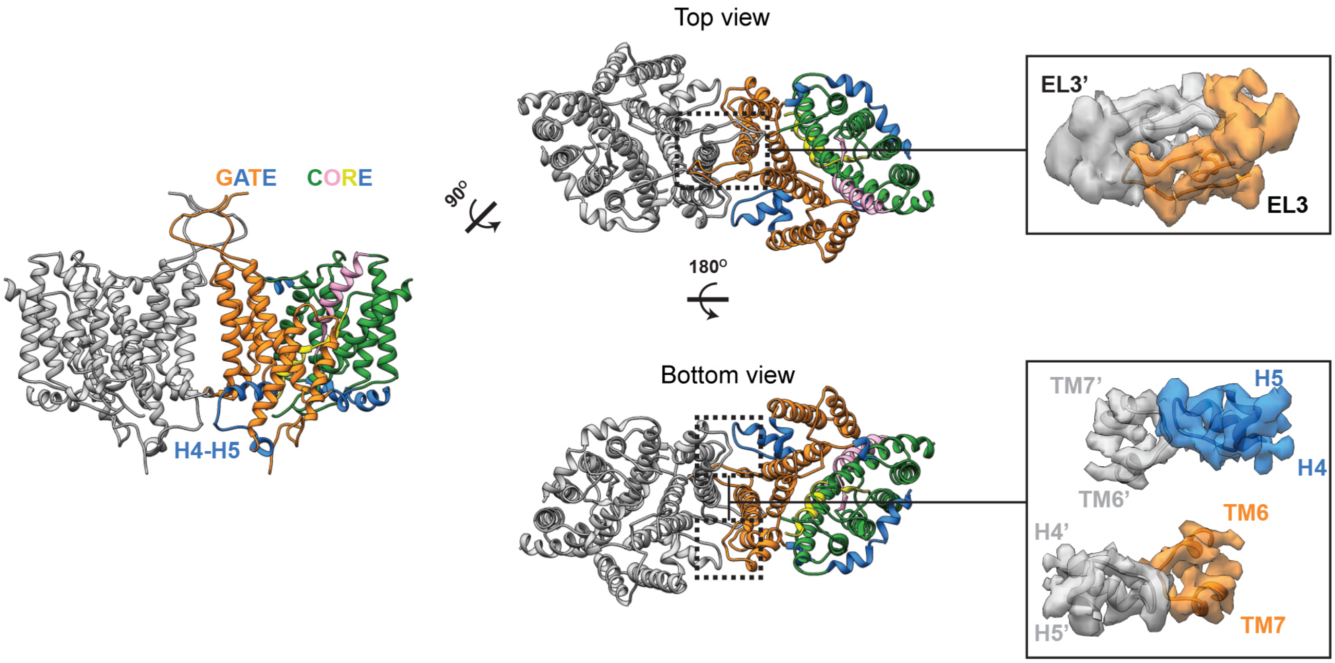

Supplementary Figure 4 NBCe1 dimer interface. Side, extracellular and cytoplasmic views of the NBCe1 model. The cryoEM densities formed by EL3 (top view) and the loops between TM6 and TM7 and H4-loop-H5 (bottom view) are shown to the right. The dimer is stabilized by interlocking EL3 loops between TM5 and TM6 and by a pair of loop-loop interactions between IL3 connecting TM6 and TM7 in one monomer and several residues between H4 and H5 in IL6 connecting TM12 and TM13 in the other monomer on the cytoplasmic side.

Supplementary Figure 5

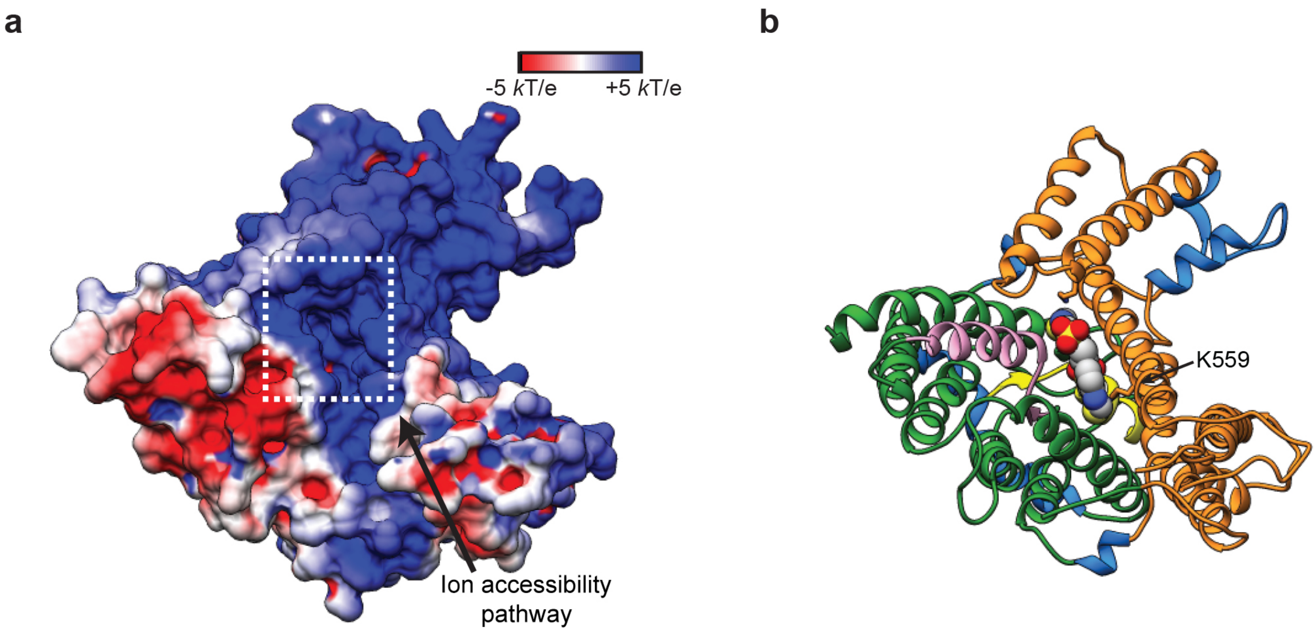

Supplementary Figure 5 Ion accessibility pathway blocked by DIDS. a Electrostatic analysis of the ion accessibility pathway (top view). **b** DIDS (shown with grey, blue and red balls) interacting with Lys559 blocks the ion accessibility pathway. We utilized previous functional inhibition data[27] and superimposed our atomic model onto the AE1 structure that was resolved in the presence of DIDS (PDB: 4YZF) using the superpose command from the CCP4 package.

Supplementary Figure 6

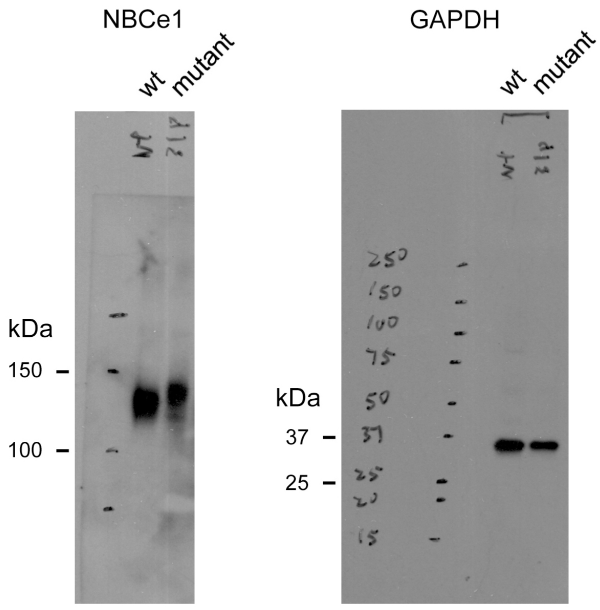

Supplementary Figure 6 Cell surface biotinylation. Immunoblot analysis of sulfo-NHS-SS-biotin labeled wt-NBCe1 and the 483SST-GFS/754D-E/798V-S/800A-T/803I-R construct (NBCe1 mutant A+B+C) expressed in HEK-293 cells.

Supplementary Table 1: Site-directed mutagenesis PCR primer sequences

Mutation	Forward primer	Reverse primer
L479C	5'- CTTTTTGCTGGTCAACCATGCACTATTCTGAGCAGCAC-3'	5'-GTGCTGCTCAGAATAGTGCATGGTTGACCAGCAAAAAG-3'
T480C	5'-CTTTTTGCTGGTCAACCACTCTGTATTCTGAGCAGCACCGGACCT-3'	5'-AGGTCCGGTGCTGCTCAGAATACAGAGTGGTTGACCAGCAAAAAG-3'
I481C	5'-TGCTGGTCAACCACTCACTTGTCTGAGCAGCACCGGACCT-3'	5'-AGGTCCGGTGCTGCTCAGACAAGTGAGTGGTTGACCAGCA-3'
L482C	5'-CTGGTCAACCACTCACTATTTGCAGCAGCACCGGACCTGTCCT-3'	5'-AGGACAGGTCCGGTGCTGCTGCAAATAGTGAGTGGTTGACCAG-3'
S483C	5'-CAACCACTCACTATTCTGTGCAGCACCGGACCTGTCCT-3'	5'-AGGACAGGTCCGGTGCTGCACAGAATAGTGAGTGGTTG-3'
S484C	5'-CACTCACTATTCTGAGCTGCACCGGACCTGTCCTAG-3'	5'-CTAGGACAGGTCCGGTGCAGCTCAGAATAGTGAGTG-3'
T485C	5'-CACTCACTATTCTGAGCAGCTGCGGACCTGTCCTAGTTTTTGA 3'	5'-TCAAAAACTAGGACAGGTCCGCAGCTGCTCAGAATAGTGAGTG-3'
V488C	5'-TCTGAGCAGCACCGGACCTTGCCTAGTTTTTGAGAGGCT-3'	5'-AGCCTCTCAAAAACTAGGCAAGGTCCGGTGCTGCTCAGA-3'
L489C	5'-GAGCAGCACCGGACCTGTCTGTGTTTTTGAGAGGCTTCTAT-3'	5'-ATAGAAGCCTCTCAAAAACACAGACAGGTCCGGTGCTGCTC-3'
F491C	5'-GACCTGTCCTAGTTTGTGAGAGGCTTCTA-3'	5'-ATAGAAGCCTCTCACAAACTAGGACAGGTC-3'
D754C	5'-CACTATACTGATTTTCATGTGCCAACAAATTACAGCTGTG-3'	5'-CACAGCTGTAATTTGTTGGCACATGAAAATCAGTATAGTG-3'
D754E	5'-TATACTGATTTTCATGGAACAACAAATTACAGCTGTG-3'	5'-CACAGCTGTAATTTGTTGTTCCATGAAAATCAGTATA-3'
T758C	5'-ATTTTCATGGACCAACAAATTTGTGCTGTGATTGTAAACAGGAAAG-3'	5'-CTTTCCTGTTTACAATCACAGCACAAATTTGTTGGTCCATGAAAAT-3'
V798C	5'-TCATGGCTCTTCCGTGGTATTGTGCTGCTACGGTCATCTCCAT-3'	5'-ATGGAGATGACCGTAGCAGCACAATACCACGGAAGAGCCATGA-3'
A799C	5'-TGGCTCTTCCGTGGTATGTATGTGCTACGGTCATCTCCATTG-3'	5'-CAATGGAGATGACCGTAGCACATACATACCACGGAAGAGCCA-3'
A800C	5'-TCTTCCGTGGTATGTAGCTTGTACGGTCATCTCCATTGCT-3'	5'-AGCAATGGAGATGACCGTACAAGCTACATACCACGGAAGA-3'
T801C	5'-CTTCCGTGGTATGTAGCTGCTTGCGTCATCTCCATTGCTCACATC-3'	5'-GATGTGAGCAATGGAGATGACGCAAGCAGCTACATACCACGGAAG-3'
V802C	5'-GTGGTATGTAGCTGCTACGTGCATCTCCATTGCTCACATC-3'	5'-GATGTGAGCAATGGAGATGCACGTAGCAGCTACATACCAC-3'
I803C	5'-GTATGTAGCTGCTACGGTCTGCTCCATTGCTCACATCGAC-3'	5'-GTCGATGTGAGCAATGGAGCAGACCGTAGCAGCTACATAC-3'
I803R	5'-GTATGTAGCTGCTACGGTCCGCTCCATTGCTCACATCGACA-3'	5'-TGTCGATGTGAGCAATGGAGCGGACCGTAGCAGCTACATAC-3'
SST(483-485)GFS	5'-GTCAACCACTCACTATTCTGGGCTTCTCCGGACCTGTCCTAGTTTTTGA-3'	5'-TCAAAAACTAGGACAGGTCCGGAGAAGCCCAGAATAGTGAGTGGTTGAC-3'
VAA(798-800)SAT	5'- TGGCTCTTCCGTGGTATTCAGCTACTACGGTCATCTCCAT-3'	5'-ATGGAGATGACCGTAGTAGCTGAATACCACGGAAGAGCCA-3'
VAATVI(798-803)SATTVR	5'-TATTCAGCTACTACGGTCAGATCCATTGCTCACATCGAC-3'	5'-GTCGATGTGAGCAATGGATCTGACCGTAGTAGCTGAATA-3'
