# Supplementary material for: CryoEM structure of the human SLC4A4 sodium-coupled acid-base transporter NBCe1
Source: Nat Commun. 2018 Mar 2;9:900. doi: 10.1038/s41467-018-03271-3 (PMC5834491; doi:10.1038/s41467-018-03271-3)
Supplement: Supplementary file 3 — Description of Additional Supplementary Files [file 41467_2018_3271_MOESM3_ESM.pdf]

Description of Additional Supplementary Files

File Name: Supplementary Movie 1
Description: CryoEM densities and atomic model of NBCe1. The cryoEM structure is rotated relative to the views of the 2D class averages (top right corner). Each monomer is color coded and the PMAL-C8 amphipol belt is represented as transparent gray. The atomic model based on the cryoEM structure is superimposed onto the cryoEM densities with the same color-coding scheme.

File Name: Supplementary Movie 2
Description: Atomic model of TM1 superimposed on the cryoEM densities. The model follows the same coloring scheme as described in Figure 1c.

File Name: Supplementary Movie 3
Description: Atomic model of the TM3 and TM10 superimposed on the cryoEM densities. The model follows the same coloring scheme as described in Figure 1c.
